# Potassium Application Improves Grain Yield and Alleviates Drought Susceptibility in Diverse Maize Hybrids

**DOI:** 10.3390/plants9010075

**Published:** 2020-01-07

**Authors:** Sami Ul-Allah, Muhammad Ijaz, Ahmad Nawaz, Abdul Sattar, Ahmad Sher, Muhammad Naeem, Umbreen Shahzad, Umar Farooq, Farukh Nawaz, Khalid Mahmood

**Affiliations:** 1 College of Agriculture, Bahauddin Zakariya University, Bahadur Sub-Campus, Layyah 31200, Pakistan; 2 Department of Plant Breeding and Genetics, University College of Agriculture & Environmental Sciences, The Islamia University of Bahawalpur, Bahawalpur 63100, Pakistan; 3 Department of Agro-ecology, Faculty of Science and Technology, Aarhus University, 8000 Aarhus, Denmark

**Keywords:** water deficit, biological yield, grain yield, drought susceptibility index

## Abstract

Maize (*Zea mays* L.) is an important component of global food security but its production is threatened by abiotic stresses in climate change scenarios, especially drought stress. Many multinational companies have introduced maize hybrids worldwide which have variable performance under diverse environmental conditions. The maize production is likely to be affected by a future water crisis. Potassium (K) is a well-known macronutrient which improves the performance of cereals under abiotic stresses. In this field experiment, we assessed the influence of soil applied K on the productivity of diverse maize hybrids grown under well-watered and drought stress conditions. The study consisted of three K levels viz., control (no KCl), KCl at 50 kg ha^−1^, and KCI at 75 kg ha^−1^ factorally combined with two irrigation levels (i.e., normal recommended irrigation, well-watered condition, and half of the recommended irrigation, drought stress condition) and eight maize hybrids. Irrigation was kept in main plots, potassium in subplot, and maize hybrids in sub-subplots. The results revealed that performance of the maize hybrids was significantly influenced by all three factors, and the interaction of irrigation with potassium and irrigation with hybrids was significant; results being non-significant for all other interactions. Potassium application improved yield traits and water productivity under both normal and water stress conditions but effect was more prominent under water stress conditions than normal conditions. Potassium application also alleviated drought susceptibility of all maize hybrids. In all cases, the performance of maize hybrids was maximum under potassium application at 75 kg ha^−1^.

## 1. Introduction

Maize (*Zea mays* L.) is the third most important cereal for global food security after wheat and rice [1,2]. Among cereal crops, maize plays a productive role in a cropping system by producing good economic return to farmers and having higher yield potential, shorter growing period, and growing ability in varying environments. It also serves as a cheaper feed source for livestock and its seeds are used in different agro-based industries. Nevertheless, in climate change scenarios, maize productivity is threatened by abiotic stresses, especially heat and drought [3,4,5,6] which in turn is threatening the global food security.

Among the abiotic stresses, drought has emerged as a serious production constraint in maize, especially in arid and semiarid regions [2,3,5,7]. The severity of water stress not only depends on the duration and intensity [8] but also on the growth stage when plants are affected, i.e., seedling, vegetative, or reproductive stage [9,10,11], all of which have differential responses but ultimately all lead to yield loss. At the early stage of water stress, losses are due to reduced growth, development, and CO_2_ fixation, while water stress at the reproductive stage leads to reproductive failure, less allocation of assimilates to the grains, and reduced grain filling period which results in shrunk grains [10,11].

Potassium is known as a stress alleviator plant nutrient which alleviates the negative consequences of abiotic stresses by regulating the physiological and biochemical process in plants [12,13,14]. Aslam et al. [12] reported that potassium improves drought tolerance by improving root growth, cell turgor pressure, and osmotic pressure. Waraich et al. [14] reported that potassium nutrition keeps a balance between antioxidant enzymes and reactive oxygen species and regulates osmotic and turgor pressure in order to avoid yield losses from drought. Potassium is also an integral part of many metabolic activities of the plants [13,15,16] and proper availability of potassium keeps the plants normal, even under drought stress, which ultimately leads to higher yield and water productivity.

Various formulations of potassium are available in the global markets. Among these, muriate of potash (KCl) is commonly used as a source of K. It contains a maximum amount of K2O, however, chloride (Cl^−^) in access amounts in KCl inhibits nitrification and affects uptake of nitrogen, potassium, phosphorus, and calcium [17] which may also result in nonsignificant effect of fertilizers on crop performance [18]. These negative consequences of Cl^−^ are more obvious in acidic soils [19,20] and are negligible in soils with high pH (≥8). Thus, application of KCl should be avoided in acidic soils. In this study, the soil had a pH > 8.0, and therefore we used KCl as a source of potassium.

A wide range of studies have investigated the impact of water stress and potassium nutrition on different field crops, including maize [12,13,15]. All crops and genotypes do not response in the same way to potassium application under all agro-climatic regions [21,22,23]. Hussain et al. [21] reported a significant interaction between mung bean genotypes and potassium application with respect to protein content and seed yield. Amanullah et al. [24] reported that foliar and soil applied potassium improved maize performance under moisture stress. Nevertheless, there is a limited research on interactive response of potassium application and maize genotypes to water stress for yield related traits and drought susceptibility index especially under arid conditions. Keeping in mind these facts, we studied the interactive response of potassium application and maize hybrids for improving drought tolerance. The specific objective of this study was to evaluate the response of diverse maize hybrids from different multinational companies to potassium application under drought stress, in terms of grain yield, water productivity, and drought susceptibility.

## 2. Materials and Methods

### 2.1. Experimental Site and Treatments

The experiment was conducted in the experimental area of the College of Agriculture, Bahauddin Zakariya University, Bahadur Sub-Campus Layyah, Pakistan. The soil of the experimental location was sandy loam with a pH of 8.1. Physiochemical properties of soil and irrigation water are presented in Table 1. The area falls in a subtropical climate characterized by warm summers and cool winters having a long-term average rainfall of ≤200 mm. Weather data of experimental duration is presented in Supplementary Table S1.

Experimental treatments include eight single cross maize hybrids (Table 2) factorally combined with three levels of potassium (control, 50 kg KCl ha^−1^, and 75 KCl kg ha^−1^) and two levels of water stress (i.e., normal irrigation of 75 mm applied every 10 days and water stress, where alternate irrigation was skipped). The maize hybrids used in study are widely grown by farmers in Pakistan. The characteristics of these maize hybrids are given in Table 2. Potassium was applied as muriate of potash (KCl). Experimental treatments were placed in a randomized complete block design with three replications and split-split plot arrangement where irrigation was kept in main plots, Potassium application was kept in subplots and maize hybrids were placed in sub-subplots.

All treatments were kept in the same plot for both years. The seed of all maize hybrids was sown by dibbler keeping an R × R and P × P distance of 45 and 15 cm, respectively, on 1 July 2017 and 2018. The nitrogen and phosphorus were applied, at the recommended rates of 170 and 115 kg ha^−1^, using urea (46% N) and diammonium phosphate (46% N, 18% P_2_O_5_) as the source. All treatments received an equal amount nitrogen and phosphors, where potassium was applied as per treatment. All fertilizers were applied on the basis of a soil analysis report. Four lines of each hybrid were sown in each replication. All the potassium and phosphorus was applied at the time of land preparation, whereas the nitrogen was applied in three equal splits (at the time of land preparation, 2 weeks after germination, and 5 weeks after germination). The total applied irrigation water was calculated as water received through irrigation and rainfall. In the well-watered condition, the total water supplied to maize crop was 600 mm, while in the drought treatment, it was 350 mm. For each rainfall event, the incident amount of rainfall was recorded through a weather station. This rainfall was subtracted from the total irrigation water applied at that interval, and the remaining amount of water was supplied through irrigation water. Cutthroat flumes were installed in the experimental plot to provide for the harvest of the calculated crop on October 20 for both years.

### 2.2. Data Collection

On maturity, data were collected for 1000-grain weight, biological yield, grain yield, and drought susceptibility index for grain yield. The 1000 grains were counted using a grain counter and weighed on an electric balance. Two central rows of each replication were harvested and oven dried until a constant weight, for the biological yield. All cobs of the same two rows were threshed to measure grain yield. Data for grain yield and biological yield was then converted into yield per hectare. Water productivity was calculated as per the following formula:

(1) $$\mathrm{Water}\;\mathrm{productivity}=\frac{\mathrm{Grain}\;\mathrm{yield}\;\left(\mathrm{kg}/\mathrm{ha}\right)\;}{\mathrm{Total}\;\mathrm{water}\;\mathrm{recieved}\;\mathrm{per}\;\mathrm{unit}\;\mathrm{area}\;\left(\mathrm{mm}\right)},$$

where total water received is water received by the crop from irrigation and rainfall. Drought susceptibility index was calculated based on grain yield as per the following formula proposed by Grzesiak et al. [25]:

(2) $${\mathrm{DSI}}_{\mathrm{GY}}=\left[1-\left({\mathrm{Grain}\;\mathrm{yield}}_{D}\times {\mathrm{Grain}\;\mathrm{yield}}_{\mathrm{IR}}^{-1}\right)\right]\times {\mathrm{DS}}^{-1},$$

where DSI_GY_ is drought susceptibility index for grain yield, (grain yield)_D_ is grain yield under drought, and (grain yield)_IR_ is grain yield under normal irrigated conditions. DS stands for drought severity index, which is calculated by the following formula:

(3) $$\mathrm{DS}=\left[{\left(\mathrm{Total}\;\mathrm{water}\right)}_{\mathrm{IR}}-{\left(\mathrm{Total}\;\mathrm{water}\right)}_{D}\right]\times {\left(\mathrm{Total}\;\mathrm{water}\right)}_{\mathrm{IR}}{}^{-1},$$

where DS is drought severity index, (total water)_IR_ is total water received by the crop under normal irrigation, and (total water)_D_ is total water received by the crop under water stress conditions. Total water received stands for cumulative water received by the irrigation and rainfall under specific irrigation treatment.

## 3. Statistical Analysis

Data collected on growth and yield parameters was analyzed statistically by using Fischer’s analysis of variance technique and HSD test at 5% probability was applied to compare the treatment means [26]. Data were analyzed using software Statistix 8.1. The year effect was found non-significant, and therefore the average of two years was used in the presentation of data.

## 4. Results

Both irrigation regimes significantly affected the 1000-grain weight, grain yield, biological yield, water productivity, and drought susceptibility index of maize hybrids. Likewise, potassium application also had a significant effect on 1000-grain weight, grain yield, biological yield, water productivity, and drought susceptibility index. Maize hybrids also differed for grain yield, biological yield, water productivity, and drought susceptibility index; results being non-significant for 1000-grain weight. The interaction of irrigation regimes with maize hybrids, and irrigation regimes with potassium application were significant for 1000-grain weight, grain yield, biological yield, water productivity, and drought susceptibility index. Two-way interaction of maize hybrids with potassium application was only significant for biological/grain yield and water productivity. All the other two-, three-, and four-way interactions were non-significant for all the studied traits (Supplementary Table S2).

Under irrigated conditions, the highest 1000-grain weight, biological yield, grain yield, and water productivity were recorded for the potassium application at 75 kg ha^−1^ and this was similar for the potassium application at 50 kg ha^−1^ for 1000-grain weight and biological yield. A similar trend was observed for potassium application under drought stress for biological yield, grain yield, and water productivity; 1000-grain weight was not significantly affected by potassium application under drought stress (Table 3).

Under well-watered conditions, the highest biological yield, grain yield, and water productivity were recorded in maize hybrid “30T60” and that was statistically similar in maize hybrid “DK-6714” for biological yield and water productivity, however, maize hybrid “DK-6714” produced significantly higher biological yield, grain yield, and water productivity under drought stress (Table 3).

The interactions showed that the highest biological yield, grain yield, and water productivity were recorded in maize hybrid “30T60” for potassium application at 75 kg ha^−1^ under well-watered conditions. Under drought stress, the biological yield, grain yield, and water productivity were higher in maize hybrid “DK-6714” for potassium application at 75 kg ha^−1^ and this was similar in maize hybrid ”30Y87” for biological yield with potassium application of 75 kg ha^−1^ (Table 3).

**Figure 1:**
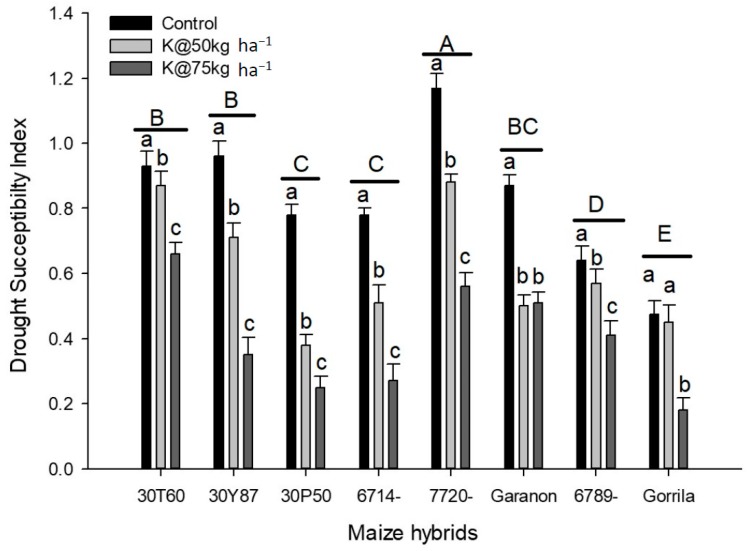
Drought susceptibility index of different maize hybrids under different treatments. Data are an average of two years and three replications. Small letters show the comparison between treatments of each hybrid and capital letters shows the comparison of different hybrids.

The drought susceptibility index was the highest in maize hybrid “S-7720” and lowest in maize hybrid “Gorrila”. The highest drought susceptibility index was recorded with potassium application at 75 kg ha^−1^ which was followed by potassium application at 50 kg ha^−1^ in all studied hybrids except the Gorilla hybrid. For the DK-Garamon hybrid, the drought susceptibility index for potassium application at 50 kg ha^−1^ was statistically similar to the control treatment (Figure 1).

## 5. Discussion

Drought stress had a negative impact on performance of all maize hybrids which was indicated through reduced values of 1000-grain weight, biological yield, and grain yield as compared with well-watered conditions. Indeed, drought has emerged as a serious production constraint in maize especially in arid and semiarid regions [2,4,5,7] which affects the crops at seedling, vegetative, and reproductive stages [9,10,11], and thus leading towards yield losses. At early-stage water stress, losses are due to reduced growth, development, and CO_2_ fixation and water stress at the reproductive stage leads to reproductive failure, less allocation to assimilate to the grains and reduced grain filling period, and less grain set which result in reduced yields [27].

However, potassium application at either rate increased the 1000-grain weight, biological yield, grain yield, water productivity, and alleviated the drought susceptibility of maize hybrids. Indeed, potassium is a stress alleviator plant nutrient which alleviates the negative impacts of abiotic stresses by regulating the physiological and biochemical process [12,13,14], including improvement in root growth, cell turgor pressure, and osmotic pressure [12], and a stable balance between antioxidant enzymes and reactive oxygen species [14].

For accumulation of dry matter in crop plants, the process of photosynthesis is of key importance, and a decrease in photosynthesis under drought stress leads to closing of stomata due to a decrease in leaf internal CO_2_ concentration and leaf transpiration rate, however, addition of K enhances photosynthesis and carbohydrate metabolism under drought stress [28,29], by improving the leaf internal CO_2_ concentration and leaf stomatal conductance which regulates the stomatal opening. The stomatal oscillations also have a strong impact on the concentration of abscisic acid in cells, and water relation of plants [30,31].

Potassium is also part of many metabolic activities of plants [13,15,16] and proper availability of potassium keeps the plant nearly normal even under drought which ultimately leads to higher yield and water productivity, as was observed in this study. Input of K improves the water relations under water stress by helping the plant absorb more water to attain turgidity. In a study, Subbarao et al. [32] found that the osmotic potential and relative water contents were decreased at low K input. Our findings of a positive role of K in alleviation of drought stress in maize can be related to the enhancement in leaf K due to an external input of K which enhances relative water contents and leaf turgor in maize [33] and sunflower [34]. Indeed, the application of K enhances the activities of different antioxidant enzymes (e.g., superoxide dismutase, catalase, and peroxidase) [35] and improves tolerance to osmotic stress. In a study, it was reported that external K input ameliorated the negative impacts of salt stress by improving the activities of antioxidant enzymes [36]. In another study, it was reported that the scavenging of reactive oxygen species through antioxidants (e.g., SOD, CAT, and GPX) can be improved by exogenous potassium application [37].

Potassium also plays key roles in sugar metabolism and sugar remobilization under drought stress. For example, Martineau et al. [38] reported that the sugars are accumulated during drought stress in maize which are reallocated with optimum potassium nutrition, possibly due to an improvement in photosynthesis and phloem transport of carbohydrates from leaves to roots. They also found that sugar transport was 35% lower in potassium deficit plants than potassium sufficient plants [38]. This decrease in assimilate transport is attributed to the decreased activity of sucrose phosphate synthase, a key enzyme in sucrose formation [39]. According to Cakmak et al. [40], the potassium effect on sucrose transport accounts for most of the plant responses undergoing potassium stress which include: sugar accumulation, negative feedback on photosynthesis [41], and lower growth rates. Potassium application also improves the zeatin, Z ribiside, and abscisic acid contents during grain filling which increase the sink strength [28,42], and thus result in better grain filling and grain yield under drought stress, as was observed in this study. Increase in grain weight due to potassium application in this study might be attributed to increased activity of photosynthesis which finally improves the source-sink relationship resulting in better grain development [24,43]. In a previous study, Amanullah et al. [24] reported that foliar and soil applied potassium improved the growth, yield, and yield components of maize under drought stress conditions. Many other studies in Pakistan [44,45,46,47], China [48,49,50,51], and USA [52] have reported an improvement in maize performance due to potassium application under optimal and suboptimal conditions. Thus, improvement in grain yield in maize hybrids under drought stress due to potassium application was possibly due to an improvement in osmolyte accumulations, activation of antioxidants, or improved sugar metabolism which improves the grain weight ultimately resulting in better grain yield and water productivity.

Maize hybrids also differed for 1000-grain weight, biological yield, grain yield, water productivity, and drought susceptibility. These differences in the grain weight, grain yield, water productivity, and drought susceptibility are desirable for cereals [53] and could be useful to identify better maize hybrids to cope with drought stress in the future. These differences are due to the diverse origin and the variation in the genetic makeup of each hybrid.

## 6. Conclusions

Potassium application improved yield traits and water productivity under both normal and water stress conditions, but effect was more prominent under water stress conditions than normal conditions. Potassium application also alleviated drought susceptibility of all hybrids. The performance of maize hybrids was maximum under potassium application at 75 kg ha^−1^, and therefore maize farmers in arid regions with a problem of water stress may get higher productions under potassium application at 75 kg ha^−1^.

## Figures and Tables

**Table 1:** Physiochemical properties of soil and irrigation water used for the experiment.

Parameter	Unit	Value		Status
		2017	2018	
Physical Parameters				
Sand	%	61.6	61.4	
Silt	%	21.9	21.3	
Clay	%	16.5	16.3	
Bulk Density	g cm^−3^	1.28	1.28	
Texture Class		Sandy Loam	Sandy Loam	
Chemical Parameters of Soil				
pH		8.1	8.1	
EC	µS/cm	1.9	1.8	
Total Nitrogen	mg kg^−1^	432	421	Very Low
Available Phosphorus	mg kg^−1^	6.9	6.8	Low
Available Potassium	mg kg^−1^	124	118	Adequate
Chemical Parameters of Water				
EC	µS/cm	309	321	
Ca+Mg	meq L^−1^	2.12	2.08	
Na	meq L^−1^	1.21	1.09	
HCO^−^_3_	meq L^−1^	1.88	1.82	
SAR	Cmolc L^−1^	1.01	0.96	

**Table 2:** Characteristics of maize hybrids used in the study.

Maize Hybrids	Source of Seed	Characteristics
30Y87	Pioneer Pakistan	Stay green character, single cross, adopted to all types of weather conditions and a yield potential of 10.4 t ha^−1^
30T60	Pioneer Pakistan	Strong stem and root, single cross, reddish seeds and a yield potential of 10.9 t ha^−1^
30R50	Pioneer Pakistan	Appropriate only for average environments
S-7720	Syngenta Pakistan	Single cross, well suited product for high density planting environments. responds very well to high management conditions with a yield potential of 9.8 t ha^−1^
DK-6714	Monsanto Pakistan	Single cross, flex ear with 117 days relative maturity and a yield potential of 11.4 t ha^−1^
DK-6789	Monsanto Pakistan	Single cross and a yield potential of 10.4 t ha^−1^
DK-Garanon	Monsanto Pakistan	White hybrid well adapted to diverse climatic conditions
Gorrila	Monsanto Pakistan	White seeded maize hybrid

**Table 3:** Influence of various potassium levels on 1000-grain weight, biological yield, grain yield, and water productivity of various maize hybrids under well-watered and drought stress conditions.

	Well-Watered				Drought			
Hybrids	K_0_	K_1_	K_2_	Mean (G)	K_0_	K_1_	K_2_	Mean (G)
	*1000-grain weight* (g)							
30T60	192.0	207.0	201.3	200.1	175.3	190.0	199.7	188.3
30Y87	181.7	194.7	207.0	194.5	186.0	194.0	196.3	192.1
30R50	185.0	188.3	196.3	189.9	173.0	180.7	165.3	173.0
DK-6714	196.7	196.3	204.0	199.0	184.7	185.7	195.7	188.7
S-7720	190.3	197.3	195.3	194.3	188.3	194.0	193.3	191.9
DK-Garanon	194.3	200.5	192.7	195.8	175.0	183.0	186.5	181.5
DK-6789	187.5	202.5	190.7	193.6	182.5	185.0	174.3	180.6
Gorrila	187.0	187.5	202.5	192.3	181.0	184.5	193.0	186.2
Mean (K)	189.3B	196.8A	198.7A		180.7	187.1	188.0	
HSD (*p* ≤ 0.05)	K = 6.7				K = 9.7			
	*Biological Yield* (t ha^−1^)							
30T60	12.3bc	12.3bc	13.4a	12.7A	9.8gh	10.3e–h	11.2a–d	10.4BC
30Y87	10.5g–j	10.7d–i	11.6c–f	10.9C	8.5i	10.3d–h	11.4ab	10.1CD
30R50	10.0h–j	10.8d–h	10.5g–j	10.4CD	8.6i	10.1f–h	10.3d–h	9.7D
DK-6714	13.1ab	12.2bc	12.4a–c	12.6A	10.3c–g	11.3a–c	11.7a	11.1A
S-7720	11.7cd	11.6c–e	11.5c–g	11.6B	9.4hi	10.2f–h	10.6b–g	10.1CD
DK-Garanon	11.1d–h	11.5c–g	12.3bc	11.6B	9.9f–h	10.7b–g	11.2a–e	10.6B
DK-6789	10.4h–j	11.1d–h	10.5f–j	10.7C	10.0f–h	10.4b–g	10.3e–h	10.2BC
Gorrila	9.7ij	10.67e–i	9.5j	10.0D	8.6i	10.2f–h	9.4hi	10.1CD
Mean (K)	11.1B	11.4A	11.5A		9.4C	10.4B	10.8A	
HSD (*p* ≤ 0.05)	G = 0.5; K = 0.3; G × K = 1.03				G = 0.5; K = 0.20; G × K = 0.90			
	*Grain Yield* (t ha^−1^)							
30T60	4.9d	5.5b	5.8a	5.4A	3.0hi	3.5d–g	4.2b	3.6B
30Y87	4.5h	4.7fg	4.8ef	4.7E	2.7ij	3.3f–h	4.1b	3.3CD
30R50	3.7l	3.8k	3.9k	3.8G	2.5jk	3.2gh	3.5e–g	3.1E
DK-6714	5.2c	5.2c	5.4b	5.3B	3.5e–g	4.1b	4.8a	4.1A
S-7720	4.3i	4.9de	5.1c	4.8D	2.2k	3.1hi	3.9bc	3.1E
DK-Garanon	4.7fg	4.8de	5.2c	4.9C	3.0hi	3.8b-e	4.1b	3.7B
DK-6789	4.1j	4.6g	4.7fg	4.5F	3.0hi	3.5efg	3.9b–d	3.5BC
Gorrila	3.5m	3.81k	3.9k	3.7G	2.9h–j	3.1gh	3.6c–f	3.2DE
Mean (K)	4.3C	4.7B	4.9A		2.9C	3.5B	4.0A	
HSD (*p* ≤ 0.05)	G = 0.06; K = 0.02; G × K = 0.13				G = 0.19; K = 0.09; G × K = 0.40			
	*Water Productivity* (kg ha^−1^ mm^−1^)							
30T60	8.2d	9.2b	9.7a	9.0A	8.6hi	10.0d–f	12.0b	10.2B
30Y87	7.5h	7.8fg	8.0ef	7.8C	7.7ij	9.4fgh	11.7b	9.6D
30R50	6.2l	6.3k	6.5k	6.3E	7.1jk	9.1gh	10.0e–g	8.8F
DK-6714	8.7c	8.7c	9.0b	8.8A	10.0e–g	11.7b	13.7a	11.8A
S-7720	7.2i	8.2de	8.5c	7.9C	6.3k	8.9hi	11.1bc	8.8F
DK-Garanon	7.8fg	8.0de	8.7c	8.2B	8.6hi	10.9b–d	11.7b	10.4B
DK-6789	6.8j	7.7g	7.8fg	7.4D	8.6hi	10.0e–g	11.1bc	9.9C
Gorrila	5.8m	6.4k	6.5k	6.2E	8.3h–j	8.9gh	10.3c–e	9.1E
Mean (K)	7.3C	7.8B	8.1A		8.1C	9.9B	11.5A	
HSD (*p* ≤ 0.05)	G = 0.18; K = 0.15				G = 0.19; K = 0.17			

K_0_: no potassium application; K_1_: 50 kg KCl ha^−1^; K_2_: 75 kg KCl ha^−1^.

## Supplementary Materials

The following are available online at https://www.mdpi.com/2223-7747/9/1/75/s1, Table S1: Weather data during the period of experimentation, Table S2: Significance terms for irrigation, potassium, maize hybrids and year on 1000 grain weight, grain yield, biological yield, water productivity and drought susceptibility index as obtained in Layyah, Pakistan.
