# Supplementary material for: Potassium Application Improves Grain Yield and Alleviates Drought Susceptibility in Diverse Maize Hybrids
Source: Plants (Basel). 2020 Jan 7;9(1):75. doi: 10.3390/plants9010075 (PMC7020434; doi:10.3390/plants9010075)
Supplement: Supplementary file 1 [file plants-09-00075-s001.pdf]

Table S1. Weather data during the period of experimentation

	Minimum temperature (°C)		Maximum temperature (°C)		Monthly rainfall (mm)	
Month	2017	2018	2017	2018	2017	2018
July	28.1	28	39.9	40.2	40	25
August	27.7	27.5	38.8	38.7	30	35
September	25	25.2	37.7	37.9	08	05
October	19.2	18.3	35	35.5	03	15

Table S2. Significance terms for irrigation, potassium, maize hybrids and year on 1000 grain weight, grain yield, biological yield, water productivity and drought susceptibility index as obtained in Layyah, Pakistan.

**Source of variance**	**1000 grain weight (g)**	**Grain yield (kg ha⁻¹)**	**Biological yield (kg ha⁻¹)**	**Water productivity**	**Drought susceptibility index**
Year (Y)	24.5^{ns}	12.9^{ns}	9.18^{ns}	12.47^{ns}	4.06^{ns}
Irrigation (I)	2316.17**	4521.43**	3247.45**	2657.12**	2794.09**
Potassium (K)	124.42**	239.22**	351.88**	249.77**	142.66**
Hybrid (H)	99.09ns	82.35**	64.02**	87.16**	106.34**
I × K	14.39**	32.42**	21.00**	36.98**	24.22**
I × H	4.72*	5.29*	2.44*	11.42**	2.99*
I × Y	0.65^{ns}	0.02^{ns}	0.35^{ns}	0.12^{ns}	0.49^{ns}
H × K	1.09^{ns}	0.45*	0.33*	1.99*	0.53^{ns}
H × Y	0.22^{ns}	0.43^{ns}	0.76^{ns}	0.27^{ns}	0.49^{ns}
K × Y	0.22^{ns}	0.66 ^{ns}	0.88^{ns}	1.02^{ns}	1.01^{ns}
I × K × H	0.42^{ns}	0.36 ^{ns}	0.77^{ns}	0.62^{ns}	0.91^{ns}
I × K × Y	0.57^{ns}	0.26 ^{ns}	0.11^{ns}	0.45^{ns}	0.66^{ns}
Y × K × H	0.71^{ns}	0.98 ^{ns}	0.29^{ns}	0.93^{ns}	1.18^{ns}
I × H × Y	0.45^{ns}	0.34 ^{ns}	0.55^{ns}	0.79^{ns}	0.06^{ns}
I × K × H × Y	0.53^{ns}	0.39 ^{ns}	0.82^{ns}	0.37^{ns}	0.52^{ns}
CV (%)	9.72	6.45	14.32	12.11	8.48

^{ns} not significant

* Significant at the 0.05 probability level.

** Significant at the 0.01 probability level.
